# Electrospun Fibrous Materials with Propolis Extracts for Edible Food Packagings

**DOI:** 10.3390/molecules28145497

**Published:** 2023-07-19

**Authors:** Zane Zelca, Remo Merijs-Meri, Andres Krumme, Aina Bernava

**Affiliations:** 1 Institute of Design Technology, Faculty of Materials Science and Applied Chemistry, Riga Technical University, LV-1048 Riga, Latvia; 2 Institute of Polymer Materials, Faculty of Materials Science and Applied Chemistry, Riga Technical University, LV-1048 Riga, Latvia; 3 Laboratory of Polymers and Textile Technology, Department of Materials and Environmental Technology, Tallinn University of Technology, 19086 Tallinn, Estonia

**Keywords:** electrospinning, nano and micro fibers, propolis, polyvinyl alcohol, food packaging

## Abstract

In this study, propolis additives provide antibacterial and antifungal effects that prolong the product’s shelf life. The aim of the study is to obtain homogeneous fiber membranes of polyvinyl alcohol and propolis by the electrospinning method and to evaluate their suitability for food packaging. Three propolis extracts are used in the study—water, ethyl alcohol, and glycerin-based. The membranes’ morphology and fiber diameter distribution, tensile deformation, air permeability, thermogravimetric analysis, differential scanning calorimetry, Fourier-transform infrared spectroscopy, and microbiological tests (*Listeria monocytogenes*, *Salmonella enteritidis*, *Escherichia coli*) were analyzed for electrospun samples. The results of the study show that propolis extracts are incorporated into membranes and the additive provides an antimicrobial effect with the contact surface. The obtained membranes are breathable: gas exchange can be controlled by using a material of appropriate thickness (air permeability coefficient is 0.046 and 0.276 mm/s). The mechanical properties of membranes are affected by moisture, but tensile strength can be improved with thermal post-processing at 100 °C. The propolis-containing fibers’ diameters are from 293 ± 8 to 664 ± 11 nm. Depending on membranes’ demonstrated properties, it can be concluded that the composites have the potential to increase the shelf life of fresh fruits and berries.

## 1. Introduction

The long-term environmental impact of plastic packaging waste is an increasing global problem. Fibrous plastic membranes with incorporated and immobilized substances (like nanoparticles, essential oils, and organic acids) with functional properties are needed to improve the properties and functionality of food packaging [1]. Waste can be reduced if the packaging can be rinsed off the food or eaten with it [2]. Preserving the freshness and flavor of food products and reduction of food waste from spoilage is an important goal that can be reached by electrospun propolis containing fibrous packaging materials. Electrospun fiber membranes can be used for food packaging directly or as a mixed electrospun packaging membrane layers, or coating with additional organic or inorganic fillers, or by using post-treatments.

Electrospun membranes could be layers to a gas barrier, and even as an emerging technology to design bioactive packaging with antimicrobial protection or delivery of supplements to foods. The main advantages of fibrous membranes are very large surface areas to volume ratios of nanofibers and high porosity [1]. Lower amounts of required active compound and good distribution are useful advantages of electrospun packaging materials, as well [3].

Recent development in active and smart food packaging focuses on biobased and biodegradable polymers with various additives (nanoparticles, plant extracts and more [4,5,6,7]). Edible food packaging can be considered a thin layer on the surface of the food product, so that pollution and waste do not occur when eaten with food, usually applied on product by spraying, dipping or wrapping [5]. Edible electrospun packaging materials are usually made of polysaccharides, proteins, lipids or combinations of them [1,8]. In this research on fibrous membrane production biodegradable polymer—Polyvinyl alcohol (PVA) also known as food additive E1203 (glazing agent, thickener)—is chosen as the fiber base material. PVA is easily electrospinnable, and widely used in food packaging, for example, as an active food packaging material layered with green tea polyphenols (with good antibacterial properties) and glycerol (as a plasticizer) [9]. PVA is water-soluble and highly impermeable to gases; therefore, it is usually used as a barrier layer for paper or packaging films [10]. Various organic acids (malic acid, tartaric acid, and lactic acid) have been added to PVA [11] to control pH, which plays an important role in achieving antibacterial properties. Essential oils are often added to electrospinning solutions as antibacterial biological additive (peppermint and chamomile essential oils) [12]. Because of cross-linking of PVA with different multi-carboxylic acid’s mechanical stability, wettability and phase morphology of PVA were enhanced [11,13]. In addition, propolis can be used as a bioactive component. The composition of propolis can vary greatly depending on where it is harvested. More than 300 compounds can be identified—aromatic acids, amino acids, phenolic compounds, essential oils, waxes and more [14]. Propolis is effective against microorganisms due to its high polyphenol content [15]. Various flavonoids are reported as effective against bacteria (*Staphylococcus aureus*, *Enterococcus faecalis* and others) and fungus (*Candida* and others) [16]. Electrospun material packaging ghas mainly been used with strawberries, grapes, tomatoes, and mushrooms, and promising results have been obtained [4].

Moreover, fresh fruits and berries are prone to fungi at all stages of their life cycle—from cultivation to the customer. Some molds can grow and produce mycotoxins on them, while some yeasts and molds can cause infections or allergies. The most common molds isolated from berries were *Botrytis cinerea*, *Rhizopus*, *Alternaria*, *Penicillium*, *Cladosporium* and *Fusarium* followed by yeasts, *Trichoderma* and *Aureobasidium*. The most common fungi on citrus fruits were *Alternaria*, *Cladosporium*, *Penicillium*, *Fusarium* and yeasts [17]. Similar microscopic fungi—*Penicillium expansum*, *Monilia fructigena* and *Neofabraea alba*—have been isolated from damaged apples stored under different conditions [18]. The *E. coli*, *Salmonella* spp. and *Listeria monocytogenes* microorganisms have also been reported on freshly cut apples [19]. Apple storage (2–4 °C, humidity 85%) and trading conditions (18 °C, humidity 65%) significantly affect the taste, hardness, pectin and phenol content and shelf life in general [18]. Packaging could play an important role in prolonging the marketing period, where fluctuations in humidity and temperature can lead to a rapid decline in fruit quality. Packaging material also requires controlled gas exchange to prevent product damage. Nanofiber-based food packaging’s air permeability characteristics allow them, especially for fruits and vegetables, to properly inhale/exhale oxygen and carbon dioxide, thus prolonging the time for which the product is fresh. Nanofiber mat pore size is small enough to retain bacteria, viruses, and other harmful pathogens, but does not completely block the airflow [20,21].

Experiments have also been performed where the air permeability properties of electrospun nanofiber materials (using PVA as one of the electrospun solution matrix) are evaluated by emphasizing the material usage as vegetable and fruit packaging material [21].

The recent developments about wrappers, bags, films and active sachets obtained through electrospinning have shown their potential application for active food packaging. There are not many packages that can prevent food spoilage while reducing microbial damage, are breathable, environmentally friendly and can be eaten with the product. Therefore, the objective of this study is to obtain homogeneous fiber membranes of polyvinyl alcohol and propolis by the electrospinning method and to evaluate their suitability for food packaging. Moreover, the suitability and effect of various propolis extracts on the properties of edible fiber membranes were evaluated.

## 2. Results and Discussion

### 2.1. Fiber Content Characterization

Depending on the propolis extract added, the fibers contain the maximum content of propolis to reach good-quality fiber membranes, shown in the Table 1. The predicted and actual amount of flavonoids analyzed in the previous study were very close [22], and the highest content of flavonoids is seen in the fibers with propolis alcohol extract which also make it possible to predict the highest efficacy against some microorganisms [16]. As can be seen in Table 1, the higher content of propolis in the fibers does not guarantee a higher content of flavonoids; this is mainly influenced by the solvent of propolis and places of propolis-harvesting origin.

### 2.2. Fourier-Transform Infrared Spectroscopy (FTIR-ATR)

**Figure 1:**
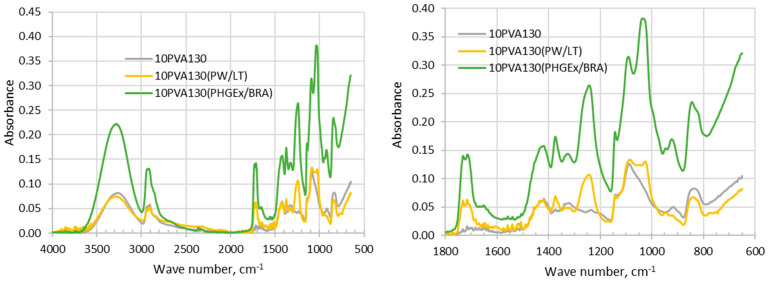
FTIR- ATR spectra of electrospun membranes of the PVA and its composites with PW/LT and PHGEx/BRA.

Analysis of FTIR-ATR spectra of the investigated electrospun membranes is challenging due to complex chemical composition of propolis, which depends upon the diversity of plants which bees have visited for gathering. It has been generally recognized that the chemical composition of propolis consists of balsam compounds (40–70%), mostly comprising numerous phenolics, and non-balsam content containing of beeswax (20–35%), essential oils (3–5%; mono and sesquiterpenes) and other organic and inorganic compounds (ca. 5%), such as polysaccharides (proteins, amino acids), mechanical impurities, etc. [23]. In the FTIR spectra of the propolis additives containing electrospun membranes (Figure 1) phenol compounds may be represented by absorption bands of O-H stretching (ca 3000–3700 cm^−1^), as well as interaction of O–H deformation and C–O stretching vibrations between 1150–1400 cm^−1^. Phenols may be also represented by vibrations at 1640–1660 cm^−1^ and 1110 cm^−1^, which have been assigned to aromatic ring C=C stretching and aromatic C–H deformation vibration respectively [23]. The absorption at 1700–1750 cm^−1^ is characteristic of the carbonyl group (C=O) stretching vibrations of the ester bond, which has been attributed to the monoesters from beeswax in propolis [24]. Absorption bands in between 1610–1699 cm^−1^ have been attributed to asymmetric bending vibrations of lipids, flavonoids and amino acids, whereas absorption bands in between 1560–1505 cm^−1^, 1450–1415 cm^−1^, 1399–1310 cm^−1^, 1198–1000 cm^−1^ were attributed to elongation, bending and stretching (C-C), bending alone (C-OH), and vibrations of flavonoids and aromatic rings, respectively [25]. Thus, vibrations centered around 1650 cm^−1^, 1370 cm^−1^, 1240 cm^−1^, 1080 cm^−1^ and 1020 cm^−1^ may be attributed to phenolics and flavonoids content in PW/LT and PHGEx/BRA. By comparing both spectra it may be concluded that flavonoids content in both propolis types is similar.

### 2.3. Fibrous Membranes Morphology

**Figure 2:**
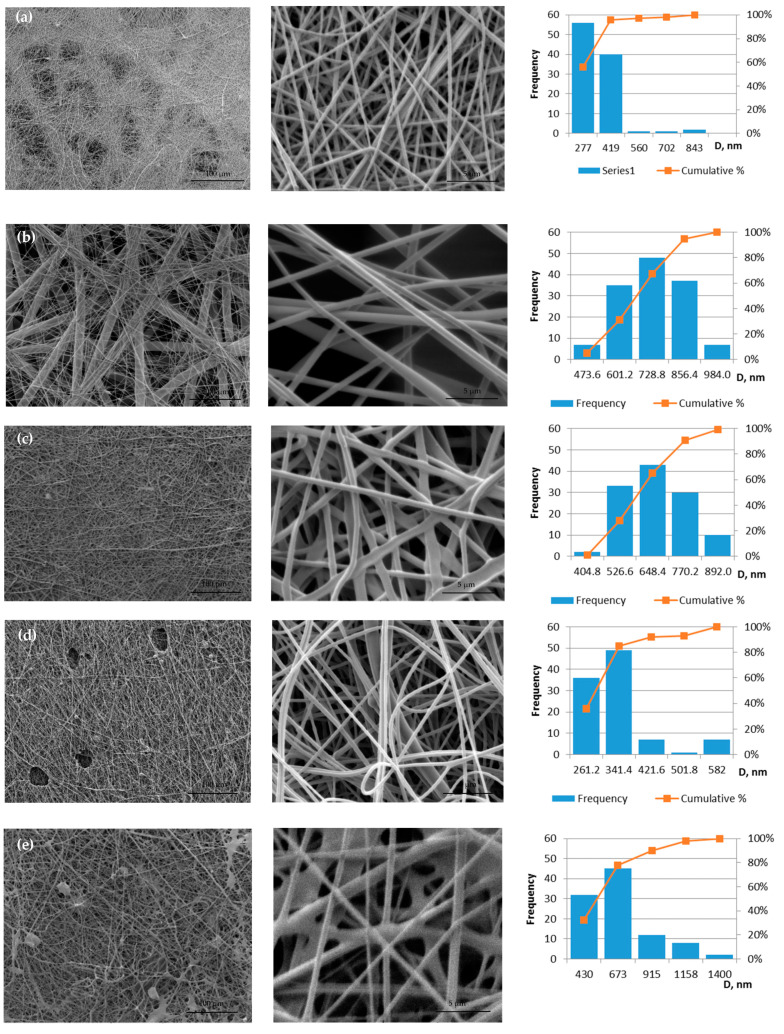
Micrographs of electrospun membranes: (**a**) 10PVA130; (**b**) 10PVA130(PW/LT); (**c**) 10PVA130(PHGEx/BRA); (**d**) 10PVA130(PEx/LV); (**e**) 10PVA145.

All prepared solutions for electrospinning are spun into fibrous membranes. The defective sample 10PVA145 is obtained using a molecular weight of PVA 145 kDa; the sample is not homogeneous: areas of film are visible (Figure 2e). The distribution of fiber diameters of two samples without propolis is shifted to the left (Figure 2 histograms a and e), but the propolis additive makes the diameter size distribution more centered (histograms Figure 2b,c), which means under the influence of propolis additive, both fiber diameters and the variety of diameter sizes increase.

The membrane of 10PVA130 (Figure 2a) has shown the fiber average diameters of 286 ± 10 nm. The addition of propolis water (Figure 2b) increased the diameter sizes to 664 ± 11 nm, hydroglyceric propolis extracts (Figure 2c) to 606 ± 11 nm [22], ethyl alcohol propolis extracts (Figure 2d) to 293 ± 8 nm. Average fiber diameters of 10PVA145 are 551± 26 nm (Figure 2e), and similar were obtained in another study—505 ± 23 nm (viscosity 1728 mPa·s) [26].

Considering that defective regions are observed in the micrographs for samples 10PVA145 and 10PVA130(PEx/LV), these samples were not further tested for tensile deformation and other tests. The results show that it is necessary to improve the spinning solution containing ethyl alcohol propolis extract and the spinning parameters may also need to be adjusted to avoid holes (Figure 2d) in the fiber membrane.

### 2.4. Differential Scanning Calorimetry

**Figure 3:**
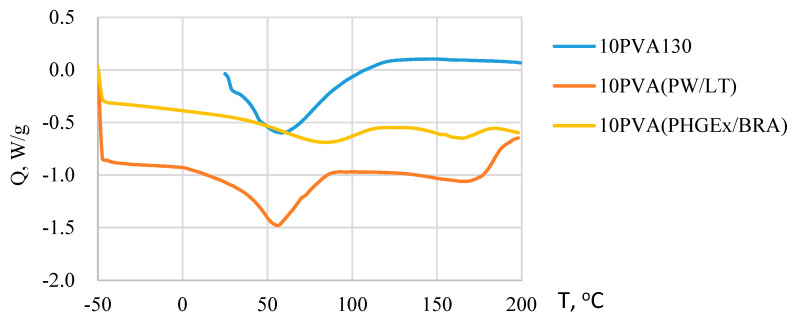
DSC thermograms of electrospun membranes of the PVA and its composites with PW/LT and PHGEx/BRA.

Due to the greatly different chemical composition of the propolis, its thermal behavior profile is complex. It has been previously demonstrated that the beeswax component of propolis undergoes various polymorphic phase transitions during melting within the temperature range ca 30–70 °C [27]. Moreover, for some propolis types melting of some natural resin components as well as removal of trapped water may occur even above 100 °C [28]. PVA melts well above 100 °C, i.e., close to 200 °C. Consequently, from DSC thermograms of electrospun PVA-propolis membranes (Figure 3) it may be concluded that within the temperature range ca 0–90 °C for 10PVA(PW/LT) and ca 35–115 °C for 10PVA130(PHGEx/BRA), the melting of propolis occurs. Concomitantly, during melting the release of water occurs. A higher melting temperature range of PHGEx/BRA most probably is connected with higher natural resin content. Endothermic peaks above 140 °C denote the beginning of thermal decomposition of active compounds, such as monosaccharides and proteins, as well as evaporation of essential oil compounds present in PW/LT and PHGEx/BRA.

### 2.5. Thermogravimetric Analysis (TGA)

**Figure 4:**
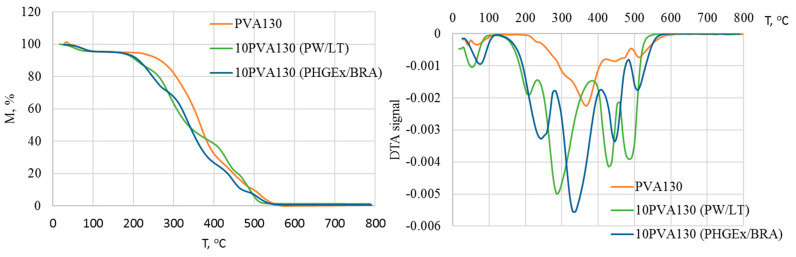
TGA and DTA thermograms of electrospun membranes of the PVA composites with PW/LT and PHGEx/BRA.

The complex chemical composition of propolis greatly influences its thermogravimetric profile, as it is demonstrated in Figure 4. Consequently, thermal degradation of the electrospun membranes may be divided into five stages. It has been previously demonstrated that in a nitrogen environment, propolis degrades via four stages: the first (90–97 °C) is attributed to release of water and unidentified low-molecular compounds; the second (100–176 °C)is attributed to decomposition of carbohydrates (mainly monosaccharides), to some extent amino acids, and aromatic and essential oil compounds, as well as some amount of trapped water; the third (200–310 °C) is attributed to the complete degradation of carbohydrates and amino acids; and the fourth (465–468 °C) major degradation stage (53–73%) is attributed mainly to the lipids [28]. Similarly, in the current case, during the first stage, at temperatures below 100 °C evaporation of water and other low-molecular compounds occurs. DTA peak maxima of 10PVA130(PHGEx/BRA) electrospun membrane is somewhat delayed, most probably due to the lower content of low molecular compounds and greater content of entrapped water in comparison to the 10PVA130(PW/LT) electrospun membrane. The second degradation stage, peaking at ca 212 °C for 10PVA130(PW/LT) and at ca 244 °C for 10PVA130(PHGEx/BRA), may be related to the degradation of carbohydrates and amino acids. The third degradation stage, peaking at 285 °C and 338 °C for 10PVA130(PW/LT) and 10PVA130(PHGEx/BRA), respectively, may be also related to the decomposition of carbohydrates and amino acids as well as their oxidation products. Peaks of DTA curves at 429 °C and 485 °C (10PVA130(PW/LT)) as well as 446 °C and 509 °C (10PVA130(PHGEx/BRA)) may be attributed to the thermooxidative degradation of amino acids and lipids, as well as their oxidation products. However, it is more plausible that the major contributant to degradation in the temperature region above ca 230 °C is PVA due to its higher thermal stability. By comparing the thermooxidative behaviour of 10PVA130(PHGEx/BRA) and 10PVA130(PW/LT) electrospun membranes, it may be concluded that the former contains larger amounts of thermally resistant constituents.

### 2.6. Microbiology Analysis of Electrospun Membranes

As expected, PVA fibers do not show antimicrobial activity against any of the microorganisms tested (Table 2). Fiber samples 10PVA130(PHGEx/BRA) and 10PVA130(PEx/LV) have a very weak effect (reduced size of microorganisms under the sample) on all microorganisms tested but sample 10PVA130(PW/LT) shows efficacy against all three microorganisms tested. The sample 10PVA130(PW/LT) shows weak growth of Listeria monocytogenes and Escherichia coli under the disk in diameter of 13 mm, but under the disc a clean zone (diameter of 13 mm) was observed against *Salmonella enteritidis* (Table 2). It can be seen that the propolis water additive has an effect against all three microorganisms: if there is contact with the surface, the microorganisms grow more slowly or not at all.

### 2.7. Tensile Stress-Strain Characteristics

**Figure 5:**
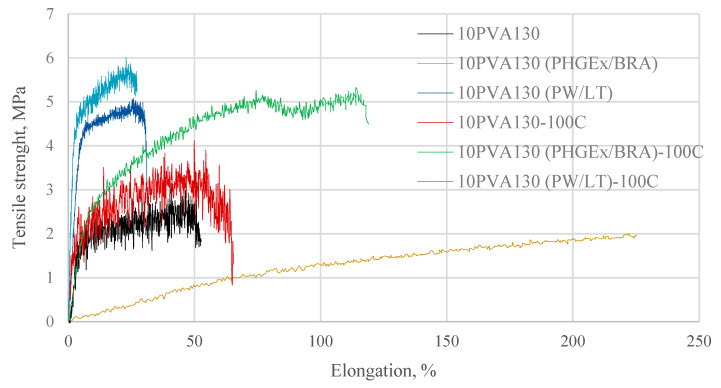
Selected stress-strain curves of untreated and thermally treated PVA composites with propolis, tested at RH = 8%.

**Figure 6:**
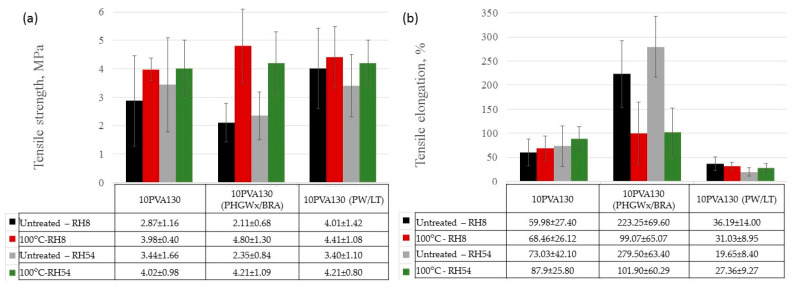
Average tensile strength (**a**) and ultimate strain (**b**) of untreated and thermally treated PVA composites with propolis, tested at different humidity levels (RH = 8% and RH = 54%).

By considering the hydrophilicity of PVA, tensile tests of the electrospun membranes have been performed at different relative humidity values (RH = 8% (dry state) and RH = 54% (moist state)). By considering great variation in the morphology (see Section 2.3), and the random fiber orientation, as well as varying of the fiber thicknesses in the obtained electrospun membranes, it has been determined that their tensile properties vary greatly. Consequently, thermal treatment/cross-linking was applied to improve the overall properties of the obtained PVA-propolis composite membranes. Thermal treatment temperature was selected to be 100 °C, at which no considerable weight loss of 10PVA130(PHGEx/BRA) and 10PVA130(PW/LT) is observed according to TGA results (Section 2.5). The results of tensile properties of untreated and thermally treated electrospun membranes of PVA and PVA with PW/LT and PHGEx/BRA are depicted in Figure 5 and Figure 6. As one can see from representative σ–ε curves of the obtained electrospun membranes, after thermal treatment the tensile strength of all the investigated electrospun membranes is increased, whereas ultimate strain is changed insignificantly or reduced to certain extent. With respect to the influence of propolis additive on the mechanical properties of electrospun membranes, it is interesting to note that after the introduction of PHGEx/BRA in the PVA matrix, the tensile strength of electrospun membranes has decreased and ultimate elongation has increased, which is opposite to the effect of introduction of PW/LT. However, this may be explained by the fact that PHGEx/BRA contains glycerine, which may act as plasticizer by reducing strength and increasing deformability of the composition. With respect to the influence of relative air moisture on the mechanical properties of the obtained electrospun membranes, it should be mentioned that in most cases ultimate elongation values of the samples tested in a moist environment are higher than those for the samples tested in dry environment. Consequently, because of the better reinforcing effect, in some cases (untreated PVA and 10PVA130(PHGEx/BRA)) tensile strength values obtained under moist environment are even higher than those obtained under dry environment.

### 2.8. Air Permeability Analysis of Electrospun Membranes

The results show that the air permeability is influenced by the following factors: additives added to the electrospun solution, fiber diameters, surface density and thickness. The air permeability of 1 µm-thick electrospun fibrous nanomembrane samples is shown in Table 3. Average results for each sample are as follows: 10PVA130—0.160 mm/s, 10PVA130(PW/LT)—0.276 mm/s, 10PVA130(PHGEx/BRA)—0.046 mm/s. Air permeability of 10PVA130(PW/LT) increases by 73%, but for sample 10PVA130(PHGEx/BRA) it decreases by 71%.

All samples show the air permeability properties, which are important for food packaging, especially for storing berries and vegetables, allowing the product to “breathe”. But it should be noted that samples with propolis water 10PVA130(PW/LT) show antibacterial activity against microorganisms, which makes this compound more suitable for food packaging.

## 3. Materials and Methods

### 3.1. Materials

PVA polymer matrix used in this research was obtained from Sigma-Aldrich Chemical Company (Darmstadt, Germany). The series of samples using PVA with different molecular weights—130 KDa (Polyvinyl alcohol 18–88, melting point is 200 °C) and 145 KDa (Mowiol 28–99, melting point is 230 °C)—were prepared. In the study, two different molecular weights of PVA were used to select the most suitable molecular weight for integrating propolis into solutions. Various types of propolis extracts were used (Table 4) as additives.

The solutions required 2–13 h of stirring at 1000 rpm on a magnetic stirrer MSH-300 (BioSan, Riga, Latvia) to prepare 60 mL of spinning solution, Table 5. In the basic mixing scheme of PVA solution, distilled water was replaced with propolis additive (6 g of PVA and 54 g of PW/LT) to prepare solution with 90 wt% propolis water (PW/LT) content according to previous research [22]. The solution with (6 g of PVA, 54 g of distilled water and 4.2 g of PHGEx/BRA) 7 wt% propolis hydroglyceric extract (PHGEx/BRA) and solution (6 g of PVA, 54 g of distilled water and 8.4 g of PEx/LV) with 14 wt% ethyl alcohol propolis extract (PEx/LV) were obtained.

### 3.2. Fabrications of Fiber Membranes

The cylinder-type electrode was used to obtain membranes by using electrospinning equipment Nanospider Lab 200 (Elmarco Company, Liberec, Czech Republic). The electrospinning voltage was 55–62 kV, the distance between the electrodes was 18 cm and the electrode rotation speed 4 rpm was adjusted according to the molecular weight of each polymer and the concentration of PVA in the solution. Electrospinning took place at 21.5 °C and relative air humidity ranged from 18–20%. Polypropylene nonwoven support material (30 g/m^2^) was used for fiber collection.

### 3.3. Characterizations of Fiber Membranes and Solutions

Fiber quality and diameters were analyzed from micrographs obtained by scanning electron microscope SEM Mira Tescan (Tescan Orsay Holding a.s., Bern, Czech Republic). Before microscopic investigations, test specimens were covered with a gold layer (5–20 nm). The image analysis program “ImageJ” was used for diameters measurements (100 measurements were obtained at 5 different locations for each sample). 

Infrared spectra in ATR mode were recorded using Thermo Fischer Scientific Fourier transform infrared spectrometer Nicolet 6700 (Thermo Fisher Scientific, Waltham, MA, USA) within the spectral range 650–4000 cm^−1^ and resolution of 4 cm^−1^.

Differential scanning calorimetry (DSC) was performed using Mettler/Toledo DSC 3 (Mettler-Toledo, LLC, Columbus, OH, USA) differential scanning calorimeter. The test was performed from room temperature to 200 °C under airflow to evaluate behavior of the developed materials during preparation and processing. Average mass of the test specimen was 10 mg.

Thermogravimetric analysis (TGA) was performed using Mettler/Toledo TGA 3+ (Mettler-Toledo, LLC, Columbus, OH, USA) thermogravimetric analyzer. The test was performed from 28 °C to 800 °C under airflow, according to ISO 11358-1:2022 [29]. Average mass of the test specimen was 10 mg.

The following cultures Listeria monocytogenes ATCC 13932, Salmonella enteritidis ATCC 13076, Escherichia coli ATCC 25922 were used for testing the antimicrobial effect. A suspension with an optical density of 0.5 McFarland (Mueller-Hinton agar or/with blood) was prepared from the microbial cultures. Then inoculation was performed on the media and the test sample (diameter 15 mm) was placed on it. After incubation (36 °C ± 1 °C, without turning with the lid up), inhibition of growth around the test material was evaluated. For quality control, standard antibiotic discs containing Ampicillin 10 µg (diameter 6 mm) were used.

Tensile properties were determined at room temperature using “Tinus Olsen TMC” universal testing machine “ST25” equipped (Tinius Olsen TMC, Horsham, PA, USA) with 1 kN load cell, according ISO 13934-1:2013 [30]. Specimens with lateral dimensions 10 × 50 mm were cut from the electrospun fibers mats and were fixed into the C-shaped paper frames. During the tensile test, prepared paper frame with test specimen was fixed in the tensile grips of the testing machine, ensuring that the distance between the grips is 20 mm, lateral border of the paper frame was cut and the test was performed at the test speed—1 mm/min. Tensile tests were performed at 2 different relative humidity levels—8% (dry state) and 54% (moist state). Part of the test specimens (20 for each sample) were tested as delivered, while others were tested after thermal treatment at 100 °C for 1 h.

Air permeability was tested using SDL Atlas Air Permeability tester (SDL Atlas Ltd., Hong Kong, China) according to LVS EN ISO 9237:2001 [31] standard. Used sample test area was 5 cm^2^ selected air pressure difference on both sides of material was 100 Pa. For each sample, 10 measurements were made in different places, and sample thickness was measured according to measured permeability area by “SDL Atlas Schrader VA-353454”. Average air permeability is expressed dm^3^/min or L/min. Coefficient of air permeability (*R*) is expressed in mm/s and can be converted to dm^3^/min or L/min to mm/s, with an Equation (1), where *q_v_*—average air permeability, dm^3^/min or L/min, *A*—sample area through which air flow is directed, cm^2^ and 167—numerical value for conversion of air permeability from dm^3^/min or L/min to mm/s.

(1) $$R=\frac{q_{v}}{A}\times 167$$

## 4. Conclusions

The results of this study demonstrated that by incorporating the propolis additives into PVA, the antimicrobial and tensile strength properties of electrospun membranes were improved. Propolis water seemed to be a more favorable additive than others for the integration of electrospun membranes due to improved tensile strength, air permeability, membrane uniformity, and antimicrobial properties. The evaluated PVA/propolis nanofibers’ tensile deformation properties change significantly under different environmental humidity conditions. To conclude, edible propolis containing PVA nano and microfiber membranes with improved functionality has the potential to be used in food-packaging applications. Further research is needed to investigate membranes’ suitability for preserving fruits and berries to obtain evidence for their potential industrial application.

## Figures and Tables

**Table 1:** Data on propolis and total flavonoids content in fibers.

Fiber Sample	Calculated Propolis Content, %	Total Flavonoids Content mg/g
10PVA130(PW/LT)	73.5	4.85 ± 0.11 [22]
10PVA130(PHGEx/BRA)	12.28	5.13 ± 0.21 [22]
10PVA130(PEx/LV)	29.58	74.51 ± 0.42 *

* Calculated Flavonoids Content mg/g.

**Table 2:** Antimicrobial activity of electrospun fibrous membranes.

Sample	Value of Inhibition Zone (mm)		
	*Listeria monocytogenes* ATCC 13932	*Salmonella enteritidis* ATCC 13076	*Escherichia coli* ATCC 25922
10PVA130	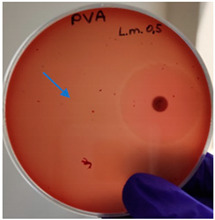	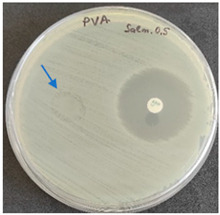	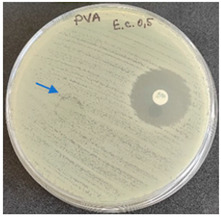
10PVA130(PW/LT)	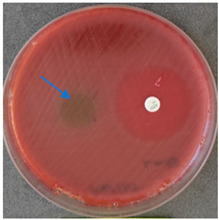	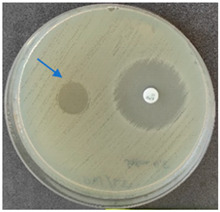	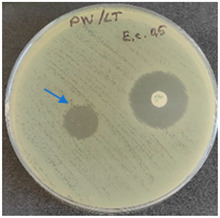

**Table 3:** Air permeability of 1 µm thick electrospun fibrous membrane samples.

Sample	Air Permeability, L/min	Air permeability Coefficient, mm/s
10PVA130	0.005 ± 0.0022	0.160
10PVA130(PW/LT)	0.008 ± 0.0023	0.276
10PVA130(PHGEx/BRA)	0.001 ± 0.0003	0.046

**Table 4:** Data on propolis and its extracts [20].

Propolis Extract	PW/LT	PHGEx/BRA	PEx/LV
Manufacturer	UAB Medicata (Vilnius, Lithuania)	B Natural SRL (Corbetta, Italy)	Laboratory-made
Propolis content	30.8%	20–25%	30%
Propolis origin	Lithuania	Brazil	Latvia
Conductivity, µS/cm	1526	3	9
pH	3.6	4.5	4.9
Total Flavonoids, mg/g	0.087 ± 0.005	1.15 ± 0.08	8.87 ± 0.05

**Table 5:** Electrospinning solutions parameters.

Sample	Molar Weight, KDa	PVA Content in Solution, wt%	Mixing Temp., °C	Propolis ex. Content, wt%	Stirring Time, h	Viscosity, mPa∗s	Conductivity, µS/cm	pH
10PVA145	145	10	90–110	0	13	782	280	5.9
10PVA130	130	10	75–90	0	2	492 [22]	366 [22]	5.8 [22]
10PVA130(PW/LT)	130	10	75–90	90	2	1004 [22]	1274 [22]	4.6 [22]
10PVA130(PHGEx/BRA)	130	10	75–90	7	3	276 [22]	290 [22]	4.8 [22]
10PVA130(PEx/LV)	130	10	75–90	14	3	170	160	5.6

## Data Availability

The data presented in this study are available on request from the corresponding author.
